# Binaural beats or 432 Hz music? which method is more effective for reducing preoperative dental anxiety?

**DOI:** 10.4317/medoral.24051

**Published:** 2020-11-28

**Authors:** Dilek Menziletoglu, Arif Yigit Guler, Tolgahan Cayır, Bozkurt Kubilay Isik

**Affiliations:** 1 Dr, Necmettin Erbakan University, Faculty of Dentistry, Oral and Maxillofacial Surgery, Konya, Turkey; 2 Specialist, Soke Oral and Dental Health Centre, Department of Oral and Maxillofacial Surgery, Aydın, Turkey; 3 Research Assistant, Necmettin Erbakan University, Faculty of Dentistry, Oral and Maxillofacial Surgery, Konya, Turkey; 4 Professor, Necmettin Erbakan University, Faculty of Dentistry, Oral and Maxillofacial Surgery, Konya, Turkey

## Abstract

**Background:**

The aim of this prospective clinical study was to investigate the effectiveness of binaural beats and music at a frequency of 432 Hz and compare which method is more effective for reducing preoperative dental anxiety in impacted third molar surgery.

**Material and Methods:**

Ninety patients were randomly selected to the binaural beats group, music group and control group. Visual analog scale used to evaluate dental anxiety before the local anesthesia in the first measurement. Local anesthesia was applied to the all patients. Patients in the music group listened to 432 Hz tuned music using earphones for 10 minutes. Patients in the binaural beats group listened to binaural beats using earphones (for the right ear, 220 Hz and for the left ear 210 Hz) for 10 minutes. No special treatment was applied to the patients in control group. In the second measurement, dental anxiety was measured again in all three groups. For analysis of differences between three groups was used One way Anova and Kruskal Wallis test.

**Results:**

Twenty seven male and 53 female patients included the study. In the first measurement, the same level of anxiety was recorded in all three groups. (*p*=0.811) There was a significant decrease in anxiety in both the binaural beats and music group in the second measurement. (*p*<0.001).

**Conclusions:**

Binaural beats and 432 Hz tuned music are a valid non pharmacological adjuvant to reduce dental anxiety in impacted third molar surgery. They have a positive effect to reduce the dental anxiety.

** Key words:**Binaural beats, 432 Hz music, dental anxiety.

## Introduction

Anxious and fearful patients are frequently encountered during the dental procedures (1,2). Although the term ‘dental anxiety’ cannot be defined exactly in the literature, it contains many different emotions ranging from mild anxiety to extreme anxiety (3). Dental anxiety is seen more common in surgical procedures (1,2). The patients being stressed during the operation reduces the patient-physician cooperation, makes the treatment difficult and may increase the stress of the physician (2). Many methods have been tried including the use of medicines to reduce the anxiety. One of them is ‘binaural beats’ technology which is based on the application of two sounds with steady intensities but different frequencies are presented separately, one to each ear. The resulting perception is of a single tone with a frequency that is midway between the two carrier tones and that waxes and wanes in amplitudes at a rate equal to the difference between them. To achieve the desired results, both ears should participate the process (4). This method has been reported to alleviate moderate anxiety (5).

Another method of reducing anxiety is music therapy. Many musicians advocated that 432 Hz is the closest frequency to the natural human frequency. Music with slow and melodies provides emotional and physical relaxation in listeners. These features make the music ‘neutral’ free from feelings that other options may trigger physiological responses in patients (3). Steelman found that music therapy reduced patients’ blood pressure under local anesthesia (6). It was reported that music intervention normalized arrhythmia and induced relaxation during local anesthesia operation (7).

Many researches about binaural beats have been done (8), but to our knowledge only one study regarding with binaural beats in dental anxiety was reported (9). In addition, there is no study comparing the effects of both binaural beats and 432 Hz music frequency on preoperative dental anxiety. The purpose of this study was to investigate the effectiveness of 432 Hz tuned music and binaural beats for reducing preoperative dental anxiety in impacted third molar surgery.

## Material and Methods

Our study’s inclusion criteria were as follows: Patients between the ages of 18-45 years; Patients who had an impacted mandibular third molar; No medication use.

Our study’s exclusion criteria were as follows: Patients with hearing disorders; Patients with psychiatric disorders; Patients who were taking antidepressants drug; Pregnant or lactating women.

We included patients referred for removal of an impacted third molar. Ninety patients were included this double-blind study between 01 January 2020 and 30 April 2020. Two researchers (AYG, TC) made the patients listen the binaural beats and 432 tuned music.

**Figure 1:**
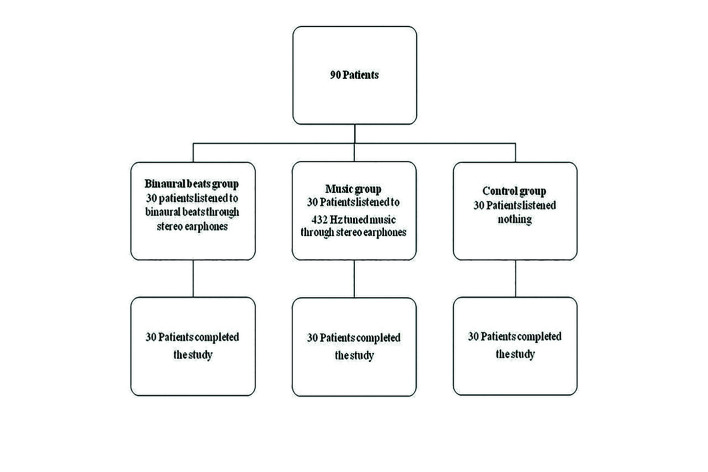
Flow diagram of the three groups for reducing preoperative dental anxiety in impacted third molar surgery.

We used Visual analog scale (VAS) to evaluate preoperative anxiety. It has been used in previous studies to evaluate dental anxiety (10,11). VAS was taken before local anesthesia from all patients in this study. This was the first measurement. VAS comprised a 100 mm horizontal line drawn on paper. The left-hand end of VAS was marked “no anxiety”, and the right-hand end “worst anxiety imaginable”. Patients were asked to mark on this line to evaluate their anxiety levels. There was no number or statement on this line. The groups were formed by taking closed envelope to the patients. The patients were divided into three groups (Fig. 1).

- Binaural Beats Group

Local anesthesia was applied to the patients. After that, the patients listened to binaural beats through stereo earphones (220 Hz for the right ear and 210 Hz for the left ear) using the mobile device (Samsung Galaxy S, Samsung Electronics Co Ltd, South Korea) for 10 minutes. The frequencies were produced by software (Brain Waves Binaural Beats, MynioTech Apps, Chapeco, Santa Catarina, Brazil) While the patients were listening to binaural beats, there was no sound in the background. The operating room in which the patients were located was quiet. The patients listened to binaural beats at the volume they wanted. We used pure frequency binaural beats without background music or relaxing sound such as waves or raindrops. During 10 minutes, we did not speak with the patients. After listening to the binaural beats for 10 minutes, the patients removed the earphones and VAS score was obtained again. It was the second measurement for the binaural beats group.

- Music Group

Local anesthesia was applied to the patients. After that, music at a frequency of 432 hz (called Summer by Stefano Crespan Shantam) was performed through stereo earphones for 10 minutes. While the patients were listening to 432 Hz music, there was no sound in the background. The patients listened to 432 Hz music at the volume they wanted. During 10 minutes, we did not speak with the patients. After listening to the music, VAS score was obtained again. It was the second measurement for the music group.

- Control Group

Local anesthesia was applied the patients and they waited the operating room for 10 minutes. Nothing was done. There was no sound or music in the operating room. After 10 minutes, VAS score was obtained again. It was the second measurement for the control group.

During 10 minutes period for three groups, the patients were told that their eyes should not be closed. Because it could affect the natural brain waves and change the results.

All patients were operated by the same oral surgeon. The operations were completed using the same local anesthetic solution, flap technique, osteotomy methods and suture materials.

- Statistical Analysis

Sigma Plot 12.5 (Systat Software Inc, San Jose, CA, USA) was used to analyze the data. Shapiro-Wilk test was used to see if the data had a normal distribution. If there was a normal distribution, paired-t test was used. If there was no normal distribution, Wilcoxon test was used. Post hoc tukey test was used after One way Anova test and Dunn’s test was used after Kruskal Wallis test to find the differences between groups. Significance level was accepted as *p* < 0.05.

## Results

Ninety patients between18-48 years of age (mean± standart deviation; 24.5± 6.49) were included. 27 of the patients were male and 53 of the patients were female. There was no statistical difference in VAS values used to measure the anxiety before the procedure (first measurement) among the three groups (*p*=0.811).

A significant decrease was found in anxiety between first measurement and second measurement in binaural beats and music group. No significant difference was observed in the control group between the first and second measurements (Table 1).

There was a statistically significant difference in the preoperative anxiety evaluation between music and control groups (*p*<0.001). There was a significant difference between binaural and control groups (*p*=0.003). There was no statistical difference between music and binaural beats group (*p*=1).

**Table 1:**
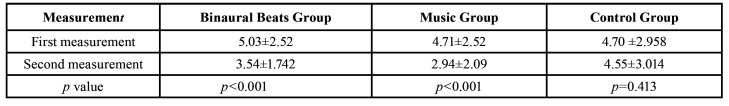
First and second VAS scores (Mean±SD) for preoperative dental anxiety in three groups.

## Discussion

The single, universal, effective method to overcome with dental anxiety is unknown (10). The administration of analgesic and sedative medications is the most preferable approach to reduce the anxiety (12). The current trend is to try to decrease anxiety by non-pharmacological intervention. Music therapy (practiced by trained music therapists) is slowly gaining acceptance as a non-pharmacological anxiolytic intervention among healthcare professionals (13). Opiate, cytokine, nitric oxide, and hormone expression in listeners can be mediated by music (14,15). Thanks to these neurochemical systems; melodic, soft and soothing music can make people quiet and relax (16). It is seen in clinical studies that music affects people’s emotions and gives positive results (16-18).

Binaural beats are also effective like music in reducing anxiety. Binaural beats were reported in 19th century and Oster (4) introduced in detail in 1973. The logic of binaural beats is to provide a sound with a constant intensity and frequency to one ear and another sound with the same intensity but slightly different frequency to the other ear. As a result, the brain produces vibrations in the amplitude and localization that is the same with the sensed sounds. These vibrations are known as ‘binaural beats’. The difference in frequency between the two sounds must be less than 30 Hz for occurring the beats, otherwise the two tones are captured independently, and the listeners can not perceive the beats (8). It has been suggested that tones with a frequency between 200 and 900 Hz are more effective than tones with exceeding 1000 Hz in provoking binaural beats (19,20).

In the meta-analysis, researchers investigated the effect of music on endoscopic procedures. They found that it had a beneficial effect in decreasing anxiety and pain (21). Song *et al* (22) prepared a review article to investigate the efficacy of the music intervention on biopsy and reported that music intervention reduced VAS pain scores after biopsy. In an endodontics study, the patients listened to 432 Hz music during their endodontic treatment. 432 Hz music has been found to reduce dental anxiety and vital signs (heart rate, systolic and diastolic pressure) (3). Isik *et al* (9) assessed effectiveness of the binaural beats in reducing preoperative anxiety in dentistry. They emphasized that binaural beats could be effective to reduce preoperative anxiety in dentistry. The study results are compatible with the literature. According to the results of our study, binaural beats and 432 Hz music are effective in reducing preoperative dental anxiety. Both methods decreased the anxiety. When two groups were compared, no statistical difference was found between them. However, the 432 Hz was found to be more effective in reducing the anxiety.

Different scales are used to assess the anxiety. However, it is known that VAS is a valid to evaluate the dental anxiety. 4.8 or more VAS scores show the dental anxiety (10). The use of the music or binaural beats reduced the dental anxiety. It was seen that waiting in the operating room without listening to 432 Hz of music or binaural beats had no positive effect on reducing anxiety.

There was only one study evaluating preoperative dental anxiety with binaural beats (9). We have taken the duration of the binaural beats as a reference. 10 minute period which we could see the effect of local anesthesia was preferred. The duration of the 432Hz music was 10 minutes in music group to ensure standardization.

Music and binaural beats are an inexpensive, safe and non-side effect method to relieve the patients. Only a smart phone, a Tablet or a personal computer are required. We recommended both methods as we reduced the preoperative anxiety of the patients.

Further studies are recommended to evaluate the variables as the duration or frequency of binaural beats and music therapy during the dental treatment.

## Conclusions

This study showed that binaural beats and 432 Hz music were effective for reducing preoperative dental anxiety in impacted third molar surgery.
